# Prevalence of sexual harassment among young Spaniards before, during, and after the COVID-19 lockdown period in Spain

**DOI:** 10.1186/s12889-022-14264-9

**Published:** 2022-10-11

**Authors:** Laura Vall-Llosera Casanovas, Laura Serra, Carme Saurina Canals, Belén Sanz-Barbero, Carmen Vives-Cases, Maria José López, Laura Otero-García, Gloria Pérez, Gemma Renart-Vicens

**Affiliations:** 1 Research Group on Statistics, Econometrics and Health (GRECS), University of Girona, C/ de la Universitat de Girona, 10 17003 Girona, Spain; 2 CIBER of Epidemiology and Public Health (CIBERESP), Madrid, Spain; 3 National School of Health. Institute of Health Carlos III, Madrid, Spain; 4 Department of Community Nursing, Preventive Medicine and Public Health and History of Science, Alicante University, Alicante, Spain; 5 Public Health Agency of Barcelona, Barcelona, Spain; 6 IIB Sant Pau, Barcelona, Spain; 7 Nursing Department. Faculty of Medicine, Autonoma De Madrid University, Madrid, Spain

**Keywords:** Sexual harassment, COVID-19, Lockdown period, Young people, Gender, sexuality

## Abstract

**Background:**

Sexual harassment is a type of coercion, including social pressure, intimidation, physical force, and verbal acts, in addition to other forms such as cyber-harassment, recognized as a major important public health problem.

**Methods:**

This cross-sectional study, based on a survey administered online to men and women aged 18 to 35 years and living in Spain throughout 15th and 28th October 2020, aims to analyze the prevalence and factors associated with sexual harassment among young people in Spain within the last 12 months, particularly according to the COVID-19 lockdown period. It has been conducted by bivariate analysis and robust Poisson regression models. The final sample includes 2.515 participants.

**Results:**

The results indicate that women were almost twice as likely as men to experience sexual harassment (49% vs 22.2%). Also, among heterosexual men and women, the estimated prevalence was lower concerning that observed among bisexuals, gays, and lesbians (31.5% vs 53, 39.2, and 34.6% respectively). The prevalence percentage in the 18–24 age group was twice high as that observed in the 30–35 age group. Finally, during the lockdown period, the harassment through electronic channels increased (32.6% vs 16.5 and 17.8% before and after this period, respectively) and decreased on public roads (22.9% vs 63.4 and 54.4% pre-lockdown and post-lockdown periods, respectively).

**Conclusion:**

These findings highlight that sexual harassment presents a high prevalence among young people, especially cyber-harassment, and workplace harassment and it is important to be aware that young women are more likely to suffer harassment and even more if they do not have a partner or have LGB orientation. During the lockdown sexual harassment has moved from public spaces to the social network.

**Supplementary Information:**

The online version contains supplementary material available at 10.1186/s12889-022-14264-9.

## Keypoints

- The incidence of SH is higher among women, young people, non-heterosexuals and those with paid work.
- The prevalence and observed prevalence estimates are higher for people who do not live with a partner or who have never had a partner compared to those who do.
- The evolution of the prevalence of SH detected before, during and after the lockdown period in Spain indicates that, for the most part, it takes place outside the home environment and public settings.
- The temporary closure of workplaces, classrooms and public environments during the lockdown period prevented face-to-face harassment, this type of conduct shifted to the digital environment.

## Introduction

Sexual violence (SV) encompasses different types of abuse that range from verbal harassment to forced penetration, as well as an array of types of coercion, from social pressure and intimidation to physical force [40]. It also includes unwanted sexual advances or sexual harassment (SH), which also ranges from physical forms through verbal acts, in addition to other forms such as cyberharassment.

The World Health Organization (WHO) defines SH as “any unwelcome sexual advance, unwelcome request for sexual favor, verbal or physical conduct or gesture of a sexual nature, or any other behavior of sexual nature that might reasonably be expected or be perceived to cause offence, humiliation or intimidation to the person” [20]. Institutions like International Labor Organization (ILO) have used similar definition with an explicit mention of the workplace or educational environments: the hierarchical and gendered power relations within occupational or educational settings have naturalized sexual contract in which some male colleagues or academics consider it a right to demand sex with female juniors or students in return for career or grades. Insistent or leering states, unwanted physical contact, sexual jokes, indecent exposure, sending sexually explicit pictures or photos, humiliations or intimidation are few examples. On the other hand, stalking is a pattern of repeated unwanted and unwarranted harassing behaviors directed by one individual in a way to cause fear, anxiety or distress: obscene, threatening, annoying or silent phone calls, being followed or spied on, having inappropriate proposals made on internet or social networks, for example [10].

U.S. Centers of Disease Control Prevention estimates that as many as 500,000 women are stalked each year by a current or former intimate partner. This number is likely to be a serious underestimate as it does not include women who are stalked by strangers or casual acquaintances [38]. Also, and according to the 2014 European FRA-Survey about Violence Against Women, one in two women (55%) in the European Union has experienced SH at least once since the age of 15, and one in five women (21%) have been subjected to this issue within the 12 months preceding the survey.

Apart from possible physical injury, stalking SH typically have effects on women’s physical and mental health. According to Kosterina et al. [21], women who had been victims of physical or sexual abuse are more likely to report dysmenorrhea, dyspareunia, migraines, gastrointestinal disorders, chronic pain, hypertension and one or more sexually transmitted diseases. Jung et al. [19] found high rates of somatic complaints such as low energy, sleep problems, headaches, muscle tension, constant fatigue, weight change, back pain, nightmares, poor appetite or trembling limbs in women who had experienced physical or sexual abuse. And these physical symptoms could persist long after the abuse was over [31]. Regarding to mental health effects and according to Brown et al. [5] and Gica et al. [17], sexual violence experiences are associated with suicidal thoughts and suicide attempts, anxiety, depression, posttraumatic stress disorders, low self-esteem and antisocial behaviors [2]. reported high average rates of alcohol, smoke and drugs abuse and eating disorders among sexual victims.

All these types of assaults suffered by women, ranging from minor injuries to death, have a significant public health and social costs. According to Sewell [36] victims of stalking and SH increase their frequency of health care utilization and the more severe the violence, the more physician visits women make. Regarding to social costs, stalking and SH it is recognized as an expression of sexism that reinforces gender-based inequalities in the distribution of social power and prevents the integration and permanence of women in work environments [29, 32].

Violence against women has been recognized as a public health problem [37] and health priority in 2013 guidelines published by WHO [16], because the number of annual injuries and death due to violence against women and girls is high enough to demand the type of active interventions and public policies [24]. Although it is a recurring public health problem over the years, recently, thanks to published information on reported cases, there is evidence that the risk of being subjected to SH among young adult women below the age of 30 is becoming a threatening matter due to its alarming prevalence (38% in the European Union) in comparison with 24% observed in older population groups. The negative social and health consequences of SH, such as depression, post-traumatic stress disorder, a feeling of being unsafe in public spaces, low self-esteem, eating disorders, and suicidal thoughts and attempts have also been registered among young adults and adolescents, with these consequences being worse in the case of women and members of the LGBT community, regardless of their migrant status or ethnic background [9, 33].

Multiple types of risk factors have been associated with SH behavior, from contextual variables, such as numerical and/or normative male dominance in universities or social media [27] to others more related with the individuals’ social circumstances, such as prior sexual victimization, hostility, alcohol consumption, and traditional gender role beliefs [25].

Coinciding with the proposal of a new national law to address SV and guarantee sexual freedom [8], this study forms part of a larger project on SV, SH, and the consumption of pornography among young adults in Spain. The data collection was carried out at the end of 2020 and served as a chance to assess the impact of the lockdown period, applied to control the spread of the 2019 coronavirus disease (COVID-19), on SH among young people and its associated sociodemographic and economic factors. Previous studies have already brought to light the increase of cases and the severity of different forms of SV among young couples and non-formal couples during the COVID-19 lockdown period [4, 11]. Although during this period personal contact between young individuals was limited, SH were easily made via social networks [18]. The aim of this study was to analyze the prevalence and factors associated with SH among young people in Spain within the last 12 months, particularly according to the COVID-19 lockdown period. According with this objective our hypotheses are the following ones: H1) People in more vulnerable and unsafe situations are more likely to suffer SH and stalking situations; H2) Youngers are more likely to suffer SH and stalking situations; H3) SH and stalking are behaviors that take place, mainly, at the workplace and at public environments; and H4) During the lockdown period, SH and stalking situations changed at the digital environment.

## Methods

### Study population and sample

This was a cross-sectional study related to the survey “Sexual violence among Young People” administered online to men and women aged 18 to 35 and living in Spain throughout the study period. The participants included in the sample were selected from a volunteer panel of 138,393 adults over the age of 16. The panel is designed to be fully representative of the non-institutionalized Spanish civilian population. The panelists received individual invitations for exclusive use inviting them to answer the survey by email. Those who accepted received a link to answer the survey. The final sample includes 2515 participants with specific quotas by sex, age, autonomous community (region) and country of birth. This sample size ensured a sampling error of ±5%, with a confidence level of 95%, and prevalence estimates with a precision of ±0.9.

The project was approved by the Ethics Committee of the University of Alicante (code UA-2020-07-07) and was carried out in accordance with the guidelines of the Declaration of Helsinki.

### Variables

#### Dependent variable

Data on SH exposure were collected based on 11 items, following national (2019 Violence Against Women Macro survey) and international guidelines [39]. Whenever a respondent answered affirmatively to having experienced any of the described actions within the last 12 months, we considered that such person had been exposed to SH. To account for the effect of COVID-19, we determined whether these SH situations had occurred before, during, or after the lockdown in Spain between 14 March 2020 and 9 May 2020.

#### Covariates

Sociodemographic, sexual orientation, and relationship covariates that have previously been correlated with SH were included in the study (see Table 1).

**Table 1:** Original covariates

Variable type	Variable	Response categories
Sociodemographic	Sex	Male Female
	Year of birth	–
	Country of birth	Spain Outside Spain
	Maximum level of education completed	Primary or lower education Secondary education completed Higher education
	Employment status	Employed Not employed
Sexual orientation	Sexual orientation	I’m exclusively attracted to women I’m usually attracted to women, but occasionally also attracted to men I’m attracted to women and men alike I’m usually attracted to men, but occasionally also attracted to women I’m exclusively attracted to men I’m not attracted to neither women nor men
Couple relationship	Currently with a partner	Yes No
	Currently living with a partner	Yes, we live at the same address Yes, but only intermittently No, we live at different addresses Another situation

Some of these original variables were recoded for the statistical analysis. The age variable was recoded into three intervals: 18 to 24 years, 25 to 29 years, and 30 to 35 years. The variable “Maximum level of education completed” was recoded into two different categories: secondary education completed and higher education, because the category primary or lower education was infrequent.

The sexual orientation variable was initially recoded into four categories according to their exclusive attraction: to same-sex ((1) gays and (2) lesbians), to opposite-sex ((3) heterosexuals), and (4) bisexuals. To include this variable in the multivariate analysis, it was grouped into two categories: lesbians, gays, and bisexuals (LGB) and heterosexuals. Respondents who were not attracted to either sex were considered missing values (*n* = 6).

The cohabitation variable was categorized into continuous cohabitation and non-cohabitation, the latter including both intermittent cohabitation and non-cohabitation. An additional category was added to both relationship variables for people who reported never having had a partner.

### Data analysis

Using the covariates described, a bivariate analysis was first carried out to determine the prevalence of SH within the last 12 months (SH12m), both among the total sample and that stratified by sex. Likewise, the prevalence of SH before, during, and after the COVID-19 lockdown period was also determined. Because the surveyed individuals could have experienced SH at any time within the previous 12 months, these three analysis periods were not mutually exclusive.

Next, we describe the prevalence of each of the 11 SH12m behaviors cited above among the total study sample and that stratified by both sex and period.

Finally, Poisson regression models with robust variance were estimated to analyze the association between the covariates and the prevalence of SH12m among the total study sample and the different analyzed periods.

Interactions between sex and the covariates included in these models were also explored. A statistically significant correlation was only observed with respect to the sexual orientation variable for the total sample and both the lockdown and post-lockdown periods. Thus, this interaction was included in all final models, except for the pre-lockdown period.

## Results

The distribution by sex and age was very homogeneous, with a majority of the participants being young people born in Spain (87.8%) and with higher level education (69.0%). Of all respondents, 61.6% claimed to be employed at the time of the survey and 78.4% had worked at some point during the previous year. The majority identified as heterosexual (75.7%) and 67.2% claimed to be in a relationship at the time, although only 38.9% cohabitated with their partner (Table 2).

**Table 2:** Sociodemographic characteristics of the participants in the “Sexual Violence among Young People” survey administered to the Spanish population aged 18–35 years

	%	N
**Age (years)**		
18–24	34.6	870
25–29	28.1	706
30–35	37.3	939
**Sex**		
Male	49.8	1253
Female	50.2	1262
**Country of birth**		
Another country	12.2	307
Spain	87.8	2208
**Education level (nc = 25)**		
Secondary education completed	31.0	771
Higher level education	69.0	1719
**Have you ever had a paid job (nc = 14)?**		
Yes, currently	61.6	1540
Yes, previously	28.4	710
No	10.0	251
**Have you had a paid job in the last year (nc = 14)?**		
Yes	78.4	1961
No	21.6	540
**Sexual orientation (nc = 38)**		
Lesbian	1.1	26
Gay	4.9	122
Bisexual	18.3	454
Heterosexual	75.7	1875
**Currently in a relationship (nc = 52)**		
Yes	67.2	1656
No	22.4	551
Never had a relationship	10.4	256
**Currently living with a partner (nc = 69)**		
Yes	38.9	951
No	28.1	688
I don’t have/never had a partner	33.0	807

Over a third of the participants (35.7%) stated having been exposed to SH within the past 12 months. The prevalence of SH was 22.2% among men and 49.0% among women. In all cases, the likelihood of SH decreased with age and was lower among participants cohabitating with a partner (27.3% vs 39.6%). In the case of women, the prevalence was higher among those who reported bisexual orientation (64.6%) and lower among lesbian participants (34.6%). In the case of men, the highest prevalence was reported by gay participants (39.2%) and lower among heterosexuals (18.3%) (see Supplementary Table S1).

Table 3 shows the significant bivariate relationships explored between SH and the analyzed covariates in each of the three periods under study. The prevalence of SH decreased from 30.4 to 11.4% during the lockdown and increased to up to 18% after the lockdown. The results indicate that the prevalence of SH12m was higher among women than men and decreased with age in all three study periods. The sexual orientation behavior showed differences in the prevalence of SH between the three analyzed periods being greater during the pre-lockdown and post-lockdown periods among bisexual participants and higher among gay participants during the lockdown period. Not having a partner was associated with a higher prevalence of this issue in all three periods, while having a job was only associated with pre-lockdown period.

**Table 3:** Bivariate relationship between the prevalence of SH and the analyzed covariates according to the study period (before, during, and after the lockdown period), based on the “Sexual Violence among Young People” survey administered to the Spanish population aged 18–35 years

	Had you ever experienced SH before the lockdown period?					Did you experience SH during the lockdown period?					Have you experienced SH after the lockdown period?				
	Yes	% of yes	No	% of no	***p***	Yes	% of yes	No	% of no	***p***	Yes	% of yes	No	% of no	***p***
**Age (years)**					**< 0.001**					0.008					**< 0.001**
18–24	**310**	**36.7**	**535**	**63.3**		110	13.1	731	86.9		**190**	**22.5**	**656**	**77.5**	
25–29	**216**	**31.2**	**476**	**68.8**		88	12.7	603	87.3		**138**	**19.9**	**554**	**80.1**	
30–35	**221**	**24.0**	**701**	**76.0**		81	8.8	840	91.2		**115**	**12.5**	**807**	**87.5**	
**Sex**					**< 0.001**					**0.002**					**< 0.001**
Male	**212**	**17.4**	**1005**	**82.6**		**114**	**9.4**	**1102**	**90.6**		**119**	**9.8**	**1099**	**90.2**	
Female	**535**	**43.1**	**707**	**56.9**		**165**	**13.3**	**1072**	**86.7**		**324**	**26.1**	**918**	**73.9**	
**Country of birth**					0.063					0.182					< 0.633
Another country	105	35.0	195	65.0		41	13.7	259	86.3		57	19.0	243	81.0	
Spain	642	29.7	1517	70.3		238	11.1	1915	88.9		386	17.9	1774	82.1	
**Education level**					0.363					0.601					0.158
Secondary education completed	217	29.0	530	71.0		81	10.9	663	89.1		122	16.4	624	83.6	
Higher level education	522	30.9	1168	69.1		196	11.6	1491	88.4		317	18.7	1375	81.3	
**Sexual orientation**					**< 0.001**					**< 0.001**					**< 0.001**
Lesbian	**7**	**26.9**	**19**	**73.1**		**3**	**11.5**	**23**	**88.5**		**4**	**4.7**	**22**	**84.7**	
Gay	**35**	**29.7**	**83**	**70.3**		**25**	**21.2**	**93**	**78.8**		**21**	**17.8**	**97**	**82.2**	
Bisexual	**202**	**46.7**	**240**	**54.3**		**80**	**18.1**	**361**	**81.9**		**126**	**28.4**	**317**	**71.6**	
Heterosexual	**496**	**27.0**	**1342**	**73.0**		**167**	**9.1**	**1666**	**90.9**		**290**	**15.8**	**1548**	**84.2**	
**Currently in a relationship**					**< 0.001**					**< 0.001**					**< 0.001**
Yes	**458**	**28.1**	**1169**	**71.9**		**147**	**9.1**	**1476**	**90.9**		**247**	**15.2**	**1379**	**84.8**	
No	**211**	**39.0**	**330**	**61.0**		**97**	**17.9**	**444**	**82.1**		**146**	**27.0**	**395**	**73.0**	
Never had a partner	**67**	**27.3**	**178**	**72.7**		**32**	**13.2**	**211**	**86.8**		**44**	**17.9**	**202**	**82.1**	
**Currently living with a partner**					**< 0.001**					**< 0.001**					**< 0.001**
No	**227**	**33.7**	**447**	**66.3**		**74**	**11.0**	**597**	**89.0**		**140**	**20.8**	**534**	**79.2**	
Yes	**223**	**23.8**	**713**	**76.2**		**72**	**7.7**	**864**	**92.3**		**104**	**11.1**	**832**	**88.9**	
Never had/does not have a partner	**278**	**35.4**	**508**	**64.6**		**129**	**16.5**	**655**	**83.5**		**190**	**24.1**	**597**	**75.9**	
**Paid work within the last 12 months**					**0.009**					0.740					0.740
Yes	**607**	**31.6**	**1311**	**68.4**		221	11.5	1693	88.5		221	18.3	1693	81.7	
No	**136**	**25.8**	**392**	**74.2**		58	11.0	468	89.0		58	17.4	468	82.6	
Total	747	30.4	1712	69.6 nc = 56		279	11.4	2174	88.6 nc = 62		443	18.0	2017	81.9 nc = 55	

The most prevalent SH behaviors reported within the last 12 months (Table 4) were the insistent or lascivious looks that have made someone feel intimidated with a 61.5% prevalence among the total sample (34.8% among men and 71.4% among women) and have received sexual jokes or offensive comments about someone’s body or private life with a 41.6% prevalence (37.4% among men and 42.4% among women).

**Table 4:** Prevalence of SH behaviors within the last 12 months according to the participants’ sex and the study period, based on the “Sexual Violence among Young People” survey administered to the Spanish population aged 18–35 years

Sexual harassment experience	Complete sample				Male				Female			
	SH12m (***n*** = 886)	Lockdown period				Lockdown period				Lockdown period		
		Before (***n*** = 747)	During (***n*** = 279)	After (***n*** = 443)	SH12m (***n*** = 273)	Before (***n*** = 212)	During (***n*** = 114)	After (***n*** = 119)	SH12m (***n*** = 630)	Before (***n*** = 535)	During (***n*** = 165)	After (***n*** = 324)
	%	%	%	%	%	%	%	%	%	%	%	%
**Have you experienced sexual harassment within the last 12 months?**	**35.7**	**30.4**	**11.4**	**18.0**	**22.2**	**17.4**	**9.4**	**9.8**	**49.0**	**43.1**	**13.3**	**26.1**
1.- Have you been subjected to insistent or lascivious glances that made you feel intimidated?	61.5	63.4	22.9	54.4	34.8	34.9	9.6	23.5	71.4	74.8	32.1	65.7
2.- Have you been shown or sent sexually explicit images or photos that made you feel offended, humiliated, or intimidated?	18.9	17.0	29.0	13.3	19.4	16.0	28.1	19.3	18.2	17.4	29.7	11.2
3.- Have you been subjected to sexual jokes or offensive comments about your body or private life?	41.6	39.7	32.2	34.3	37.4	39.6	24.6	27.7	42.4	39.8	37.6	36.7
4.- Have you ever been made inappropriate suggestions to have a date or any type of sexual activity that made you feel offended, humiliated, or intimidated?	20.9	17.7	21.5	19.9	15.0	12.3	14.0	18.5	22.8	19.8	26.7	20.4
5.- Have you been subjected to unwanted physical contact, such as unnecessarily close proximity, touching parts of your body, kissing/hugging, or any other type of contact that you did not want?	25.9	25.0	9.7	18.7	19.4	16.5	11.4	18.5	28.1	28.4	8.5	18.8
6.- Have you received inappropriate, humiliating, intimidating, or offensive advances via social networks such as Facebook, Instagram, or Twitter?	23.6	20.1	35.1	20.1	24.2	20.8	33.3	27.7	22.7	19.8	36.4	17.3
7.- Have you received sexually explicit or inappropriate emails, WhatsApp messages, or text messages that made you feel offended, humiliated, or intimidated?	19.4	16.5	32.6	17.8	23.8	22.6	34.2	26.9	16.9	14.0	31.5	14.5
8.- Have you been threatened with unpleasant consequences at work, such as a layoff, loss of salary supplements, continuous criticism of your work, etc. if you refused sexual advances?	1.4	1.1	0.7	0.9	2.6	2.4	1.8	1.7	0.8	0.6	0.0	0.6
9.- Has anyone indecently exposed themselves to you?	9.5	8.7	5.0	6.5	14.6	13.2	6.1	14.3	6.9	6.9	4.2	3.7
10.- Have you been forced to watch pornographic material against your will?	2.2	1.9	3.9	1.3	5.1	4.2	6.1	4.2	0.9	0.9	2.4	0.3
11.- Other than those listed above, have you experienced any similar situation with a sexual connotation that made you feel offended, humiliated, or intimidated?	7.9	7.1	5.4	5.6	4.8	4.7	3.5	4.2	9.0	8.0	6.7	6.2

The described patterns were maintained during the pre-lockdown and post-lockdown periods; however, during the lockdown, the most frequent behaviors were to have received inappropriate, humiliating, intimidating or offensive insinuations on social networks such as Facebook, Instagram or Twitter with a 35.1% prevalence (33.3% among men and 36.4% among women) and have received sexually explicit and inappropriate e-mails, WhatsApp messages or text messages that have someone feel offended, humiliated or intimidated with a 32.6% prevalence (34.2% among men and 31.5% among women). In contrast, behaviors whose prevalence clearly decreased during the lockdown period are also worth noting, including have had unwanted physical contact with unnecessarily close proximity, touching parts of someone’s body, kisses/hugs or anything else not wanted with a 9.7% prevalence (11.4% among men and 8.5% among women) and the insistent or lascivious looks that have made someone feel intimidated with a 22.9% prevalence (9.6% among men and 32.1% among women).

Table 5 shows the results of the multivariate estimation of the prevalence ratios among the total sample throughout the different periods. Within the last 12 months, women were 84.7% more likely to be sexually harassed than men (95% CI; PR [1.54, 2.22]), with this issue being particularly prevalent among those with a paid job (95% CI; PR [1.18, 1.56]), who did not cohabitate with a partner (95% CI; PR [1.16, 1.56]), or who had never had a partner (95% CI; PR [1.36, 1.79]) compared with those who were in a relationship. In contrast, the likelihood of suffering SH was 23.7% lower among participants of the 30–35 age group compared with those aged up to 25 years (95% CI; PR [0.66, 0.88]) P. The interaction between sex and sexual orientation showed that heterosexuals are less likely to suffer SH than LGB, specifically 43.0% less in the case of men and 22.79% less in women.

**Table 5:** Variables associated with sexual harassment within the last 12 months before, during, and after the lockdown period: Poisson regression with robust variance

Variables	SH12m OR (95% CI)	Lockdown period		
		Before OR (95% CI)	During OR (95% CI)	After OR (95% CI)
Sex (male)				
Female	**1.847 (1.537, 2.220)**	**2.521 (2.192, 2.898)**	0.982 (0.696, 1.385)	**1.939 (1.441, 2.609)**
Age (25 years old or younger)				
Between 25 and 29 years	0.897 (0.791, 1.016)	**0.851 (0.736, 0.983)**	1.055 (0.790, 1.409)	0.975 (0.799, 1.189)
Between 30 and 35 years	**0.763 (0.660, 0.882)**	**0.726 (0.615, 0.857)**	0.846 (0.613, 1.168)	**0.716 (0.564, 0.908)**
Country of birth (Spain)				
Outside Spain	1.149 (0.997, 1.324)	1.117 (0.944, 1.321)	1.301 (0.960, 1.763)	1.094 (0.862, 1.388)
Education level (secondary education completed)				
Higher level education	1.038 (0.921, 1.171)	1.002 (0.888, 1.169)	1.052 (0.808, 1.370)	1.078 (0.887, 1.310)
Paid job within the last 12 months (no)				
Yes	**1.357 (1.180, 1.562)**	**1.492 (1.264, 1.760)**	1.302 (0.962, 1.762)	**1.272 (1.027, 1.577)**
Cohabitation with a partner (yes)				
No	**1.343 (1.158, 1.557)**	**1.280 (1.082, 1.514)**	**1.417 (1.009, 1.989)**	**1.654 (1.287, 2.125)**
Never had a partner	**1.557 (1.356, 1.787)**	**1.488 (1.271, 1.741)**	**2.147 (1.578, 2.921)**	**2.207 (1.748, 2.787)**
Sexual orientation (LGB)				
Heterosexual	**0.570 (0.461, 0.704)**	**0.719 (0.638, 0.810)**	**0.376 (0.265, 0.533)**	**0.462 (0.329, 0.649)**
Sexual orientation * sex (LGB male)				
Heterosexual woman	**1.356 (1.067, 1.723)**		**1.971 (1.248, 3.114)**	**1.766 (1.193, 2.613)**

Shaded, the 95% credibility interval did not contain the unit (i.e. statistically significant at 95%)

The same relationships described within the last 12 months were observed during both the pre and post lockdown periods, with the only difference that the likelihood of suffering SH was also lower among participants of the 25–29 age group compared with younger individuals before the lockdown period (95% CI; PR [0.74, 0.98]), although no correlation between sex and sexual orientation was identified. These relationships changed the most during the lockdown period, as the effect of sex, work status, and age on the likelihood of suffering SH was lost, although a greater likelihood was still maintained among young individuals who did not cohabitate with a partner or who had never had a partner compared with those who did (95% CI; PR [1.01, 1.99] vs. 95% CI; PR [1.58, 2.92]). Finally, the interaction between sex and sexual orientation allows us to affirm that, during the lockdown period, heterosexual participants were less likely (62.4 and 25.93% for men and women, respectively) to experience harassment than participants pertaining to the LGB community.

## Discussion

The results of our analysis clearly show that SH is a manifestation of power that primarily disadvantages women, younger individuals, non-heterosexuals, and those with a paid job.

Our results indicate that women are almost twice as likely as men to experience SH. While men can also be subjected to SH, women are more frequently disempowered because of a lack of physical and economic strength, being in more vulnerable and unsafe situations, and suffering the consequences of a heteropatriarchal education and cultural system, so the sexual harasser is usually a man and the harassed person tends to be a woman [13, 22].

We observed that, among heterosexual men and women, the estimated prevalence of SH was lower with respect to that observed among the LGB community, with prevalence estimates in the bivariate analysis of 31.5% for heterosexuals compared with 53, 39.2, and 34.6% for bisexuals, gays, and lesbians, respectively. Other studies also suggest that non-heterosexuals experience a higher percentage of harassment behaviors compared with heterosexuals [1, 3]. These authors also indicate that the most common negative effects of cyberbullying on LGB youth are psychological, emotional and behavioral, causing more problems in the sports world due to fear of being bullied and also showing lower academic performance.

Coinciding with the literature (2019 Violence Against Women Macrosurvey), our study shows that age is also a key factor in the incidence of SH, with the estimated prevalence being higher among younger individuals compared with older ones, with a prevalence percentage in the 18–24 age group twice as high as that observed in the 30–35 age group.

Employment status is another factor that showcased these power structures as we found that the likelihood of SH is higher among people who have a paid job compared with those who do not. Conversely, no significant differences were observed among men with respect to this variable. This finding make it clear that SH is present in the workplace and that the harasser is often a person belonging to the work environment, such as a colleague, a boss, or a client ([34]; 2019 Violence Against Women Macrosurvey).

In a more intimate environment, SH can also take place in a relationship context. However, our statistical model yielded higher prevalence estimates for people who did not cohabitate with a partner or who had never had a partner with respect to those who did. Although harassment within a relationship was normalized for several years due to the belief that this was a private matter, it has repercussions on the victims’ health and an important social impact [12]. Harassment within a relationship between two young individuals who do not cohabitate or are not married is clearly different to that occurring within the context of a marriage or cohabitating partners. First, because of the harassers’ and the victims’ age, which is markedly lower among non-cohabitating partners. Secondly, due to factors related to parental, contractual, or financial responsibilities in the case of cohabiting couples [30]. Thus, the higher prevalence of SH among individuals without a partner or who do not cohabitate with a partner may be explained by their age (which tends to be higher among people with a partner) or the fact that harassment occurring within a relationship takes on a dimension of sexual violence that is experienced and reported by the victim as such.

The evolution of the prevalence of SH detected before, during, and after the lockdown period indicates that this type of harassment, for the most part, takes place outside the home environment: in the work environment, the academic environment [7, 28] and in public settings (street SH) [35]. Thus, the disappearance of sex, age, and paid work as variables affecting SH during the lockdown period would be explained by the temporary closure of workplaces, classrooms, and both public (squares, streets, etc.) and private (bars, restaurants, pubs, etc.) social spaces.

Although the temporary closure of workplaces and classrooms prevented face-to-face harassment, this type of behavior entered the digital environment through offensive messages, insinuations or proposals, provocations, contact attempts using false identities, messages with sexual content or offensive calls being just some of the SV acts mostly experienced by women and teenagers [6]. Sexual cyberstalking is a crime contemplated in the amendment of the 2015 Spanish penal code. Therefore, we are describing acts that have only been criminalized since very recently. It is important to highlight variables such as gender, a couple relationship, age, country of birth, and sexual orientation within the context of technological harassment. As in the case of other studies, our analysis highlights that women, usually in a stable relationship [26], are the main victims of these digital aggressions (66.7% compared with 33.3% among men) [23]. These cyber-aggressions among couples is a subtype of phycological aggression that takes place via social media aimed to threaten, humiliate and control partner behaviour and social relationships [14]. Moreover, as shown by our findings, the most vulnerable groups (minors, immigrants, or the LGTBI community) suffer more from this type of harassment [15].

The above is evident in the findings of our study. The most frequently reported SH behaviors before and after the lockdown periods were insistent or lascivious looks that have made someone feel intimidated (63.4 and 54.4% pre-lockdown and post-lockdown periods, respectively, versus 22.9% during the lockdown period) and have received sexual jokes or offensive comments about someone’s body or private life (39.7 and 34.3% pre-lockdown and post-lockdown periods, respectively, versus 32.2% during the lockdown period). In contrast, during the lockdown period, harassment through electronic channels, such as have received inappropriate, humiliating, intimidating or offensive insinuations on social networks such as Facebook, Instagram or Twitter (35.1% during the lockdown period versus 20.1% both before and after this period) or have received sexually explicit and inappropriate e-mails, WhatsApp messages or text messages that have someone feel offended, humiliated or intimidated (32.6% during the lockdown period versus 16.5 and 17.8% before and after this period, respectively), was more significant, which highlights the fact that the harasser was an acquaintance who had the harassed person’s contact details.

The results obtained in our analysis are consistent with those reported in existing publications in the scientific literature but provide new insights into SH regarding the use of a mixed sample (comprising both women and men). This approach avoids the gender bias affecting many studies including exclusively female samples by justifying that only women suffer this type of harassment. However, this claim falls apart when considering non-heterosexual men. Our sample design and the breadth of the panel used to extract it allowed us to determine the exposure to SH among young Spaniards.

Although much work is being carried out to raise society’s awareness of harassment, there is still a long way to go. We live in a society that is currently making great progress in certain aspects; however, there is still discrimination based on a person’s gender, sexual orientation, or age supported by power relations that fail to respect equal opportunities. That’s why our results are relevant, because they can help to design preventive policies and programs that can be applied in adolescents and youth populations to prevent these behaviors in the future. Because the eradication of SH (as well as other types of abuse) will require a cultural change influencing political, commercial, and educational formulations throughout different countries to ensure that equal opportunities are truly given to all people, regardless of their age, race, gender, or religion.

## Supplementary Information

**Additional file 1: Table S1.** Prevalence of SH according to the study covariates of the “Sexual Violence among Young People” survey administered to the Spanish population aged 18–35 years.

## Data Availability

The datasets generated and analysed during the current study are not publicly available because we are still carrying on research papers and the project is not already finished. However, they are available from the corresponding author on reasonable request.
