# Supplementary material for: Prevalence of sexual harassment among young Spaniards before, during, and after the COVID-19 lockdown period in Spain
Source: BMC Public Health. 2022 Oct 11;22:1888. doi: 10.1186/s12889-022-14264-9 (PMC9551249; doi:10.1186/s12889-022-14264-9)
Supplement: Supplementary file 1 — Additional file 1: Table S1. Prevalence of SH according to the study covariates of the “Sexual Violence among Young People” survey administered to the Spanish population aged 18–35 years. [file 12889_2022_14264_MOESM1_ESM.docx]

**Supplementary table**

**Table S1. Prevalence of SH according to the study covariates of the “Sexual Violence among Young People” survey administered to the Spanish population aged 18-35 years**

|  | **Total sample** | | | |  | | **Male** | | | | |  | | **Female** | | | | |  |
| --- | --- | --- | --- | --- | --- | --- | --- | --- | --- | --- | --- | --- | --- | --- | --- | --- | --- | --- | --- |
|  | **% of yes** | **% of no** | **total** | ***p*** | |  | | **% of yes** | **% of no** | **total** | ***p*** | |  | | **% of yes** | **% of no** | **total** | ***p*** | |
| **Age (years)** |  |  |  | **<0.001** | |  | |  |  |  | **0.011** | |  | |  |  |  | **<0.001** | |
| 18-24 | **42.7** | **57.3** | **853** |  | |  | | **26.3** | **73.7** | **418** |  | |  | | **58.4** | **41.6** | **435** |  | |
| 25-29 | **37.2** | **62.8** | **697** |  | |  | | **23.0** | **77.0** | **348** |  | |  | | **51.3** | **48.7** | **349** |  | |
| 30-35 | **28.3** | **71.7** | **929** |  | |  | | **18.0** | **82.0** | **462** |  | |  | | **38.5** | **61.5** | **467** |  | |
| **Sex** |  |  |  | **<0.001** | |  | |  |  |  |  | |  | |  |  |  |  | |
| Male | **22.2** | **77.8** | **1228** |  | |  | | 22.2 | 77.8 | 1228 |  | |  | |  |  |  |  | |
| Female | **49.0** | **51.0** | **1251** |  | |  | |  |  |  |  | |  | | 49.0 | 51.0 | 1251 |  | |
| **Country of birth** |  |  |  | **0.033** | |  | |  |  |  | 0.396 | |  | |  |  |  | 0.272 | |
| Another country | **41.3** | **58.7** | **303** |  | |  | | 25.2 | 74.8 | 127 |  | |  | | 52.8 | 47.2 | 176 |  | |
| Spain | **35.0** | **65.0** | **2176** |  | |  | | 21.9 | 78.1 | 1101 |  | |  | | 48.4 | 51.6 | 1075 |  | |
| **Education level** |  |  |  | 0.232 | |  | |  |  |  | 0.657 | |  | |  |  |  | 0.433 | |
| Secondary education | 34.0 | 66.0 | 753 |  | |  | | 22.9 | 77.1 | 410 |  | |  | | 47.2 | 52.8 | 343 |  | |
| Higher level education | 36.5 | 63.5 | 1704 |  | |  | | 21.8 | 78.2 | 807 |  | |  | | 49.7 | 50.3 | 897 |  | |
| **Sexual orientation** |  |  |  | **<0.001** | |  | |  |  |  | **<0.001** | |  | |  |  |  | **<0.001** | |
| Lesbian | **34.6** | **65.4** | **26** |  | |  | | **__** | **__** | **__** |  | |  | | **34.6** | **65.4** | **26** |  | |
| Gay | **39.2** | **60.8** | **120** |  | |  | | **39.2** | **60.8** | **120** |  | |  | | **__** | **__** | **__** |  | |
| Bisexual | **53.0** | **47.0** | **445** |  | |  | | **31.8** | **68.2** | **157** |  | |  | | **64.6** | **35.4** | **288** |  | |
| Heterosexual | **31.5** | **68.5** | **1851** |  | |  | | **18.3** | **81.7** | **933** |  | |  | | **44.9** | **55.1** | **918** |  | |
| **Paid work within the last 12 months** |  |  |  | 0.062 | |  | |  |  |  | 0.527 | |  | |  |  |  | **0.013** | |
| No | 32.3 | 67.7 | 532 |  | |  | | 20.8 | 79.2 | 250 |  | |  | | **42.6** | **57.4** | **282** |  | |
| Yes | 36.7 | 63.3 | 1934 |  | |  | | 22.7 | 77.3 | 975 |  | |  | | **51.0** | **49.0** | **959** |  | |
| **Currently in a relationship** |  |  |  | **<0.001** | |  | |  |  |  | **<0.001** | |  | |  |  |  | **<0.001** | |
| Yes | **32.6** | **67.4** | **1639** |  | |  | | **18.4** | **81.6** | **759** |  | |  | | **44.9** | **55.1** | **880** |  | |
| No | **45.5** | **54.5** | **543** |  | |  | | **29.6** | **70.4** | **284** |  | |  | | **62.9** | **37.1** | **259** |  | |
| **Never had a partner** | **35.7** | **64.3** | **249** |  | |  | | **26.9** | **73.1** | **160** |  | |  | | **51.7** | **48.3** | **89** |  | |
| **Currently living with a partner** |  |  |  | **<0.001** | |  | |  |  |  | **<0.001** | |  | |  |  |  | **<0.001** | |
| No | **39.6** | **60.4** | **681** |  | |  | | **22.9** | **77.1** | **323** |  | |  | | **54.7** | **45.3** | **358** |  | |
| Yes | **27.3** | **72.7** | **941** |  | |  | | **15.1** | **84.9** | **431** |  | |  | | **37.6** | **62.4** | **510** |  | |
| Never had/does not have a partner | **42.4** | **57.6** | **792** |  | |  | | **28.6** | **71.4** | **444** |  | |  | | **60.1** | **39.9** | **348** |  | |
| **Total harassment within the last 12 months** | **35.7** | **64.3** | **2474**  **nc = 36** |  | |  | | **22.2** | **77.8** | **1228**  **nc = 25** |  | |  | | **49.0** | **51.0** | **1251 nc = 11** |  | |
